# Cytogenetic identification and molecular marker development of a novel wheat-*Leymus mollis* 4Ns(4D) alien disomic substitution line with resistance to stripe rust and Fusarium head blight

**DOI:** 10.3389/fpls.2022.1012939

**Published:** 2022-11-01

**Authors:** Xin Du, Xianbo Feng, Ruoxuan Li, Yanlong Jin, Lihui Shang, Jixin Zhao, Changyou Wang, Tingdong Li, Chunhuan Chen, Zengrong Tian, Pingchuan Deng, Wanquan Ji

**Affiliations:** ^1^ College of Agronomy, Northwest A & F University, Yangling, Shaanxi, China; ^2^ State Key Laboratory of Crop Stress Biology for Arid Areas, Northwest A & F University, Yangling, Shaanxi, China; ^3^ Shaanxi Research Station of Crop Gene Resources and Germplasm Enhancement, Ministry of Agriculture, Yangling, Shaanxi, China; ^4^ Institute of Crop Sciences, Chinese Academy of Agricultural Sciences, Beijing, China

**Keywords:** *Leymus mollis*, alien disomic substitution line, stripe rust, FHB, transcriptome sequencing, liquid array

## Abstract

*Leymus mollis* (Trin.) Pilg. (2*n* = 4*x* = 28, NsNsXmXm) potentially harbours useful genes that might contribute to the improvement of wheat. We describe M862 as a novel wheat-*L. mollis* alien disomic substitution line from a cross between wheat cv. 7182 and octoploid *Tritileymus* M47. Cytological observations indicate that M862 has a chromosome constitution of 2*n* = 42 = 21II. Two 4D chromosomes of wheat substituted by two *L. mollis* Ns chromosomes were observed, using the GISH and ND-FISH analyses. Molecular marker, 55K SNP array and wheat-*P. huashanica* liquid array (GenoBaits^®^WheatplusPh) analyses further indicate that the alien chromosomes are *L. mollis* 4Ns. Therefore, it was deduced that M862 was a wheat-*L. mollis* 4Ns(4D) alien disomic substitution line. There were also changes in chromosomes 1A, 1D, 2B and 5A detected by ND-FISH analysis. Transcriptome sequencing showed that the structural variation of 1D, 1A and 5A may have smaller impact on gene expression than that for 2B. In addition, a total of 16 markers derived from Lm#4Ns were developed from transcriptome sequences, and these proved to be highly effective for tracking the introduced chromosome. M862 showed reduced height, larger grains (weight and width), and was highly resistance to CYR32 and CYR34 stripe rust races at the seedling stage and mixed stripe rust races (CYR32, CYR33 and CYR34) at the adult stage. It was also resistance to Fusarium head blight (FHB). This alien disomic substitution line M862 may be exploited as an important genetic material in the domestication of stipe rust and FHB resistance wheat varieties.

## 1 Introduction

Wheat is the most extensive and largest cultivated cereal worldwide, providing a variety of nutrients and about 20% of the calories consumed by humans (Shewry and Hey, 2015). Wheat production is threatened by various biotic and abiotic stresses, whereas beneficial genetic materials are increasingly scarce. Wild relatives have numerous agronomically valuable genes that can be introduced into common wheat by distant hybridization (Du et al., 2013; Liu et al., 2019), serving as an important way to broaden the genetic base of modern wheat varieties (Sepsi et al., 2008; Wang et al., 2020c).

Stripe rust (YR) caused by *Puccinia striiformis* f. sp. *tritici* (*Pst*) is a severe economic disease resulting in several pandemics and significant production losses worldwide (Yaniv et al., 2015; Sha et al., 2016a). At present, 88% of wheat yield around the world is threatened by stripe rust, and surpasses one billion dollars per year (Beddow et al., 2015). Fusarium head blight (FHB), caused by *Fusarium graminearum*, is emerging as a widely distributed and devastating disease to global wheat production (Wang et al., 2020). Deoxynivalenol (DON) is the most common and toxic fusarium mycotoxin, as well as the crucial virulence factor of *F. graminosa* infection (Chen et al., 2019). The rapid evolution and spread of new virulent wheat stripe rust races (Zhan et al., 2015), as well as limited FHB resistance sources, promote characterization and deployment of wild relatives to overcome these threats (Wang et al., 2020b).

*Leymus mollis* (Trin.) Pilger (2*n* = 4 *x* = 28, NsNsXmXm), a perennial tetraploid species in *Leymus mollis* (Gramineae: Myaceae), is mainly distributed in the northern coastal areas of China, Russia (Far East), North Korea, Japan and North America (Fan et al., 2009). It possesses strong and short stems, large spikes, multiple spikelets, tolerance to drought, cold, alkaline and saline conditions, in addition to resistance to many fungal and bacterial diseases (stripe rust, powdery mildew, leaf rust and Fusarium head blight) (Fatih, 1983; Mujeeb-Kazi et al., 1983; Merker, 1992), serving as an important tertiary gene pool for wheat breeding (Kishii et al., 2003). *Leymus* genome composition is generally regarded as NsNsXmXm. It has been proved that Ns subgenome in *Leymus* originated from *Psathrostachys* (Zhang and Dvôrák, 1991; Zhang et al., 2006; Fan et al., 2009; Culumber et al., 2011; Sha et al., 2016a; Sha et al., 2017a). Meanwhile, the origin of the Xm subgenome is still controversial, which had been proposed to be donated by *Agropyron* (P), *Eremopyrum* (F) (Fan et al., 2009; Sha et al., 2017a) or *Psathyrostachys* (Ns) (Sha et al., 2017a) or vary among species (Sha et al., 2016a).

Since the 1960s, wheat and *L. mollis* hybridization had been applied successfully (Tsitsin, 1965). The first hybrid seeds were obtained by embryo rescue and colchicine treatment of the hybrid between common wheat 7182 and *L. mollis* in China (Chen et al., 1985). The derivatives of wheat and *L. mollis* have appeared successively. Octoploid *Tritileymus* (2*n* = 8x = 56, AABBDDNsNs or AABBDDXmXm) displayed excellent agronomic characteristics, such as large grain and panicle, many flowers, and resistance to various fungal diseases (Wang et al., 2000; Wang et al., 2012). A series of addition, substitution, and translocation lines were obtained by crossing durum wheat or common wheat with octoploid *Tritileymus* (2*n* = 56, AABBDDNsNs) (Bao et al., 2012; Zhao et al., 2013; Pang et al., 2014a; Pang et al., 2014b; Zhao et al., 2016; Zhao et al., 2019). Several wheat-*L. mollis* addition lines and substitution lines with resistance to stripe rust and powdery mildew using a cross between common wheat 7182 and partial amphiploid M47 (2*n* = 8x = 56, AABBDDNsNs) were created previously (Yang et al., 2014a; Yang et al., 2015; Yang et al., 2017; Zhang et al., 2017a; Yang et al., 2020). Furthermore, a few studies have reported that the useful trait on the resistance to FHB for *L. mollis* have been introgressed into cultivated wheat varieties (Chen et al., 2005; Qi et al., 2008; Zhao et al., 2019).

Molecular marker analysis is the most convenient method to identify and trace the alien genetic material in the common wheat background (Wang et al., 2020c). The main markers include simple sequence repeats (SSRs), expressed sequence tag (EST), and PCR-based Landmark Unique Gene (PLUG) (Du et al., 2013; Pang et al., 2014a; Yang et al., 2020). However, these markers have some disadvantages such as poor stability and repeatability, as well as low specificity. Currently, high-throughput sequencing technology has been widely applied in the development of wild relatives specific markers as their high-efficiency and low-cost, including SLAF-seq (Chen et al., 2013; Zhang et al., 2015; Liu et al., 2018; Song et al., 2020; Wang et al., 2020c), GBS (Li et al., 2019a) and transcriptome sequencing (Li et al., 2017). So far, only two studies have reported the development of markers tightly linked to *L. mollis*, including 14,530 SNP markers developed based on genome DArTseq (Edet et al., 2018) and 13 DNA markers developed for 2Ns chromosome by SLAF-seq (Feng et al., 2022).

It is far from enough to introduce the exogenous genetic material from *L. mollis* into the background of wheat. Therefore, researchers need to continuously create distant hybridization materials of wheat and *L. mollis* with excellent disease resistance or other beneficial traits. In this study, a novel wheat-*L. mollis* 4Ns(4D) alien disomic substitution line M862 was obtained from a cross between common wheat 7182 and octoploid *Tritileymus* M47. M862 was analyzed by cytological identification, genome *in situ* hybridization (GISH), fluorescence *in situ* hybridization (FISH), wheat 55K SNP array, wheat-*P. huashanica* liquid array and molecular marker (SSR, EST, PLUG) identification, as well as disease resistance and agronomic traits evaluation. The results demonstrated that the wheat-*L. mollis* 4Ns(4D) alien disomic substitution line showed high resistance to stripe rust and FHB. Meanwhile, we develop specific molecular markers of Lm#4Ns, which will improve the efficiency of wheat breeding.

## 2 Materials and methods

### 2.1 Plant materials

Common wheat line 7182 (2*n* = 6x = 42, AABBDD), Huixianghong (HXH), Sumai3 (SM3), Xiaoyan22 (XY22), Chinese Spring (CS), Aikang58 (AK58), wheat-related species of *Leymus mollis* (Trin.) Pilger (2*n* = 4*x* = 28, NsNsXmXm), *Psathyrostachys huashanica* Keng ex P.C.Kuo (2n = 2x = 14, NsNs), *Thinopyrum ponticum* (Podp.) Barkworth & DR Dewey (2*n* = 10*x* = 70, E^e^E^e^E^b^E^b^E^x^E^x^StStStSt or JJJJJJJ^s^J^s^J^s^J^s^), *Secale cereale* L. (2*n* = 2*x* = 14, RR) and *Hordeum vulgare* L. (2*n*=2*x*=14, HH), the progeny of wheat-*L. mollis* M47 (2*n* = 8*x* = 56, AABBDDNsNs), alien disomic substitution lines of Lm#1Ns(1D) and Lm#4Ns(4D), disomic addition lines of Lm#2Ns, Lm#3Ns, Lm#5Ns, Lm#6Ns and Lm#7Ns were used in this study (**Supplementary Table 1**). Among them, Lm#4Ns(4D) line M862 was derived from the BC_1_F_4_ progeny of a 7182/M47//M47 cross. M47 was a partial amphidiploid derived from common wheat 7182 and *L. mollis* (Yang et al., 2014a).

### 2.2 Cytological identification

For mitosis analyses, root tips were collected from ten independent healthy plants grown in the field during late March over two growing seasons (2021 and 2022, five plants per year). The excised roots were immediately placed on ice for 24 h. Subsequently, the roots were rinsed thoroughly with water to remove soil, then fixed in 3:1 Carnoy’s fluid (3:1 ethanol: glacial acetic acid) at room temperature. After fixation for 48 h, the root tips were stained with 1% acetic acid magenta for 12 h and stored at 4°C until further use. After removing the root caps from well-stained root tips, 1 mm of the meristematic zones were immersed in a drop of 45% acetic acid on a clean slide for cytogenetic analysis.

For meiosis analyses, young spikes were excised from ten healthy plants grown in the field during early April over two growing seasons (2021 and 2022, five plants per year), and fixed in 6:3:1 Carnoy’s fluid (6:3:1 ethanol: chloroform: glacial acetic acid) for 48-72 h (room temperature). Then, the anthers were stored at 4°C) until further use. The fixed anthers were squashed on slides and stained with 1% aceto-carmine before examination. Homologous chromosomes pairing in pollen mother cells were observed by Olympus BX43 microscope (Tokyo, Japan).

### 2.3 GISH, sequential FISH-GISH and ND-FISH analysis

Root tip processing procedures were performed as previously described (Han et al., 2006). The genomic DNA of *L. mollis* and *P. huashanica* were purified and used as probes by labeling with fluorescein-12-dUTP and Texas Red-5-dCTP. GISH was used to detect the alien chromosome composition of M862 (Zhang et al., 2017a). Sequential FISH-GISH was performed to analyze the karyotypes of M862 and foreign chromosomes on the same slide. The probes of Oligo-pSc119.2 (6-FAM-5′, green), Oligo-pTa535 (Tamra-5′, red), and Oligo-D (6-FAM-5′, green) were synthesized by Invitrogen Biotechnology Co., Ltd (Shanghai) (Tang et al., 2018), which were further used to detect the chromosome composition of D subgenome in M862 as previously described (Tang et al., 2014). 4, 6-diamidino-2-phenylindole (DAPI) was used to counterstain chromosomes. The slides were examined with the Olympus BX63 fluorescence microscope equipped with a DP80 camera (Tokyo, Japan).

### 2.4 Homoeologous group identification

#### 2.4.1 Wheat 55K SNP Array and Wheat-*P. huashanica* Liquid Array Analysis

Genomic DNA of M862, 7182, and *L. mollis* were hybridized to Wheat 55K SNP genotyping array by China Golden Marker Biotechnology Company (Beijing). There were 53007 microarray probe sites per chip, including diploid markers. Based on IWGSC-RefSeq-v1.0 (https://wheat-urgi.versailles.inra.fr/SeqRepository/), a total of 49,060 markers had specific physical location information, evenly covering the whole wheat genome. The average distance between the markers was 0.2~0.3Mb. A dish quality control (DQC) value ≥ 0.82 and call rate (CR) ≥ 90 were the thresholds used as the criteria for SNP filtering. Collected data were analyzed using an Excel V2019 and Origin V2019 (OriginLab, USA).

Additionally, a wheat-*P. huashanica* liquid array (GenoBaits^®^WheatplusPh) was further used to clarify the alien chromosomes homoeologous group of M862. Genomic DNA of M862 was sent to MolBreeding Biotechnology Company (Shijiazhuang, China) for DNA library preparation and probe hybridization, which was further sequenced on the Illumina HiSeq X. The sequencing data were first checked for quality control, then aligned to the reference genome using BWA (Li, 2012). The sequencing depth for each probe was calculated by bamdst v.1.0.6 (https://github.com/shiquan/bamdst), then plotted using R package.

#### 2.4.2 Molecular makers analysis

122 SSR makers, 34 EST-STS markers (http://wheat.pw.usda.gov/SNP/new/pcr_primers.shtml) and 13 PLUG (Polymerase Chain Reaction-based landmark unique gene) markers (Hu et al., 2011) were used to further determine the homoeologous group of the alien chromosomes. All primers were synthesized by Beijing AuGCT DNA-SYN Biotechnology Co., Ltd. As previously described (Yang et al., 2014a), 8% non-denaturing polyacrylamide gel was used to separate the PCR products of EST-STS and PLUG markers. PCR products of PLUG makers were digested with *HaeIII* at 37°C for 3 h or *TaqI* at 65°C for 2 h (Zhang et al., 2017a).

### 2.5 Disease resistance assessment

The *Pst* races CYR32 and CYR34 were used to assess stripe rust resistance at the seedling stage, while the mixed races (including CYR32, CYR33, and CYR34) were used to identify stripe rust resistance at the adult stage. At the seedling stage, 10 plants per pot were inoculated with CYR32 and CYR34 *Pst* races using spraying method in three independent biological repeats. After inoculation, the seedlings were firstly placed in a humidor that was kept in the dark for 24 hours at 8°C. Then, the incubator parameters were adjusted to 14h in the daytime, 60% humidity, 6000Lx, and 8 h at night with 70% humidity. When the control material HXH was fully infected, we began to assess the disease resistance every 4 days until 21 days after inoculation (Wang et al., 2020c).

Adult stage stripe rust resistance trials were performed under field conditions over two growing seasons (2021 and 2022) using mixtures of *Pst* races (CYR32, CYR33 and CYR34). All materials were planted in two rows with 20 plants. At the jointing stage, the mixed *Pst* races and talcum powder were inoculated by shaking powder method in a ratio of 1:200 after spaying water onto plants (Wang et al., 2020c). When the disease of HXH was completely developed, the symptoms of the infected plants were recorded, again 7 days later. Immunity type (IT) was divided into 6 grades from 0-4 (IT 0, IT 0;, IT 1, IT 2, IT 3, and IT 4) as previously reported (Ma et al., 1995). In brief, IT 0 = immune, no visible uredia and necrosis on leaves; IT 0; = nearly immune, no uredia with hypersensitive flecks on leaves; IT 1 = very resistant, few small uredia clearly visible embedded in well-defined necrotic areas; IT 2 = moderately resistant, few small- to medium-sized uredia surrounded by clearly-defined but less extensive necrotic areas; IT 3 = moderately susceptible, a lot of medium-sized uredia, no necrosis, but with chlorosis on leaves; IT 4 = highest susceptible, a large number of large-sized uredia without necrosis on leaves.

M862 and its parents were used to assess FHB resistance over two growing seasons (2021 and 2022) in the field. *F. graminearum* strain PH1 was used to evaluate FHB resistance. SM3 and XY22 were used as susceptible and resistant controls for FHB, respectively. At the flowering stage, 10 spikes were selected from each material in the field. The 5th floret from the top of each selected spike was inoculated by injecting 10 μL micro-conidial suspension (250,000 spores ml^-1^). Afterward, transparent plastic bags sprayed with mist water were placed on the injection spikes and kept at a high temperature until 72 h after inoculation. Disease severity in the inoculated wheat spikes was assessed 21 days after inoculation (Guo et al., 2015). The infected spikelet rate (ISR, %), the severity level (SL, 0 to 4) and resistance evaluation (RE, I, R, MR, MS, S) of FHB were recorded based on the Agricultural Industry Standard of the People’s Republic of China (PRC, 2007). FHB Type II resistance was defined as suppressing the spread of the disease from the point of infection.

### 2.6 Agronomic traits assessment

M862 and its parents were evaluated for the following agronomic traits: plant height, tiller number, spike length, spikelets number, florets number per spikelet, kernel number per spike, 1000-kernel weight, kernel length, kernel width and awnedness. Ten plants were measured for each material over two growing seasons (2021 and 2022). The length and width of grain were performed on 200 to 250 grains per line using the SC-G automated seed testing system (Wseen Detection Technology Co., Ltd., Hangzhou, China) (repeated five times). Data were tested for statistical significance by using the IBM SPSS Statistics 23 software (IBM Corp., Armonk, NY, USA).

### 2.7 Transcriptome sequencing and data analysis

RNA was extracted from the fresh leaves (third leaf stage) of M862 and 7182 using TRIZOL reagent (Thermo Fisher Scientific Inc., Shanghai, China) according to the manufacturer’s instructions. RNA quality was assessed using Agient 2100 Bioanalyzer. Transcriptome sequencing was performed using an Illumina NovaSeq 6000 platform at Beijing Biomarker Technologies Corporation (Beijing, China), with three biological replicates for each material. Clean data were obtained by removing the reads containing adaptor, poly-N and low quality using FASTX-Toolkit (Schmieder and Edwards, 2011). High-quality clean data from each sample were separately aligned to the wheat reference genome (IWGSC RefSeq v1.1) by Hisat2 (Pertea et al., 2016) (**Supplementary Figure 1**). To construct the candidate unigene dataset for Lm#4Ns, the unmapped reads for all samples were used in *de novo* transcriptome assembly using Trinity software package (Grabherr et al., 2011) with default parameters. The high-quality clean data from each sample were aligned to wheat reference sequences and *de novo* assembly by Hisat2 (Pertea et al., 2016). Only uniquely mapped reads were used for gene-level quantification using StringTie (Pertea et al., 2016). Differentially expressed genes (DEGs) were calculated by DESeq2 (FPKM≥1, Log_2_|fold change|≥1, adjusted *p ≤* 0.05). Unigenes expressed in M862 but not expressed in 7182 were retained as Lm#4Ns candidate genes. To further evaluate the Lm#4Ns gene, the sequences were used to search against the existing protein database of Swiss-Prot, *Triticum urartu* (A), *Aegilops speltoides* (B), *Aegilops tauschii* (D), *Hordeum vulgare* (H), *Secale cereale* (R), *Thinopyrum elongatum* (E), *Aegilops longissima* (S). GO enrichment analysis was performed by AgriGO v2.0 analysis tools (http://bioinfo.cau.edu.cn/agriGO/). A corrected *p ≤* 0.01 was set as a threshold to determine the significant enrichments.

### 2.8 Lm#4Ns-specific marker development based on the RNA−seq data

The Lm#4Ns candidate unigenes that meet the following criteria were used to develop molecular marker: (I) unigene sequences that had the highest identity to the fourth group chromosome in at least one related species; (II) a sequence identity less than 50% by comparison with the wheat reference genome sequence (IWGSC-RefSeq-v1.1). (III) more than 50% by comparison with the *P. huashanica* Keng reference genome sequence (unpublished). Primers were designed in batches using the primer3_core program (Deng et al., 2016). The major parameters for primer designing were set as follows: 18-27 bp in primer length, 57-63°C in melting temperature, 40-60% in GC content and 200-700 bp in product size. The designed primers were searched against the genomes of wheat, barley and rye on WheatOmics 1.0 (http://wheatomics.sdau.edu.cn/), and sequence-free amplification was finally used to synthesize the primers at AuGCT (Beijing AuGCT Biotechnology Co. Ltd., Beijing, China). Markers were firstly screened in M862 and its parents, then verified in the common wheat (CS and AK58), wheat-related species (*P. huashanica*, *Th. ponticum*, *S. cereale* and *H. vulgare*), and wheat-*L. mollis* derived progenies (Lm#1Ns(1D), Lm#2Ns, Lm#3Ns, Lm#5Ns, Lm#6Ns and Lm#7Ns).

## 3 Results

### 3.1 Cytological characterization of M862

**Figure 1:**
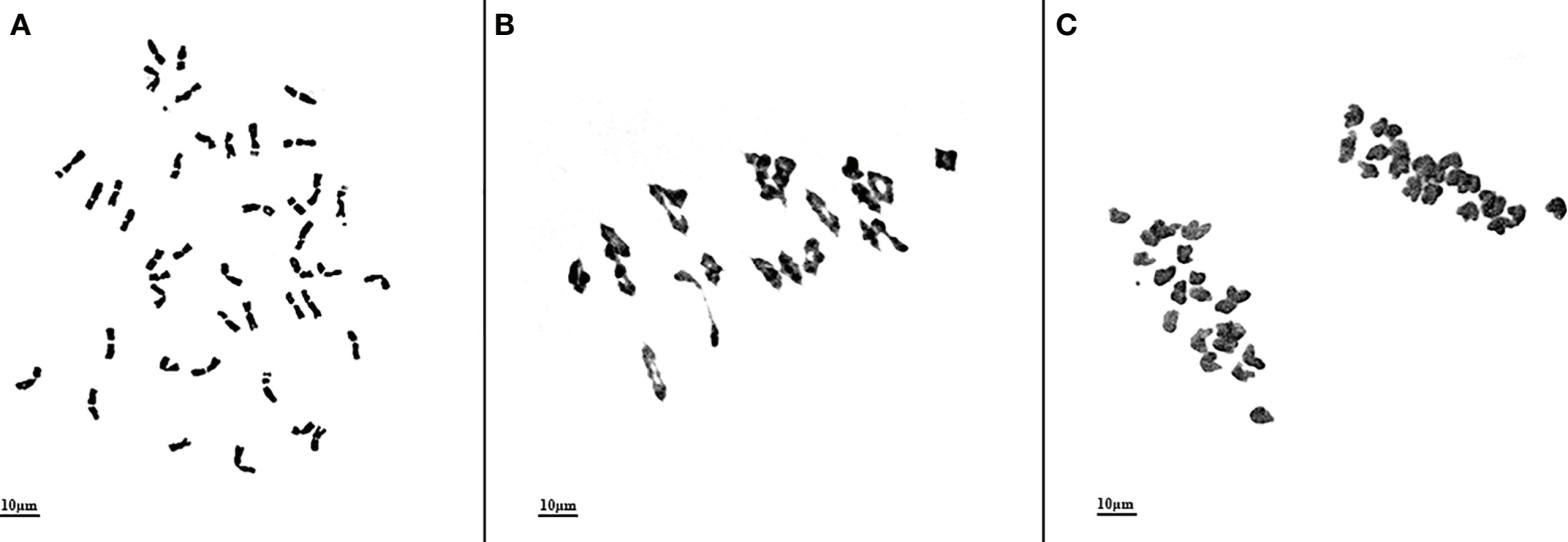
Cytological observations of wheat-*Leymus mollis* substitution line M862. **(A)** Chromosomes of root tip cell at mitotic metaphase, 2*n* = 42. **(B)** Chromosome characteristics of pollen mother cell at meiotic metaphase I, 2*n* = 21II. **(C)** Chromosome characteristics of pollen mother cell at meiotic anaphase I, 2*n* = 21’ + 21’. Scale bar = 10 μm.

M862 was derived from the BC_1_F_4_ progeny of the cross between common wheat cultivar 7182 and wheat-*L. mollis* partial amphidiploid M47 (2*n* = 56). The mitotic observation of M862 showed that 156 out of 161 cells (96.89%) in 28 root tips from ten plants (2021 and 2022, five plants per year) had a chromosome number of 2*n* = 42 (**Figure 1A**). There were 2 and 3 cells with 41 and 40 chromosomes, respectively. The meiotic analysis of 117 pollen mother cells (PMCs) at metaphase I (MI) in two years showed that 110 (94.02%) PMCs had a chromosome configuration of 2*n* = 21 II (**Figure 1B**), and 7 PMCs had 2*n* = 20 II + 2 I. While no trivalents or quadrivalents were observed at MI, and no chromosomes lagged at anaphase I. 21’+21’ form was produced by equal separation of chromosomes at anaphase of meiosis II (**Figure 1C**). Cytological observations indicated that M862 was a highly stable line.

### 3.2 GISH, sequential FISH-GISH and ND-FISH analyses of M862

**Figure 2:**
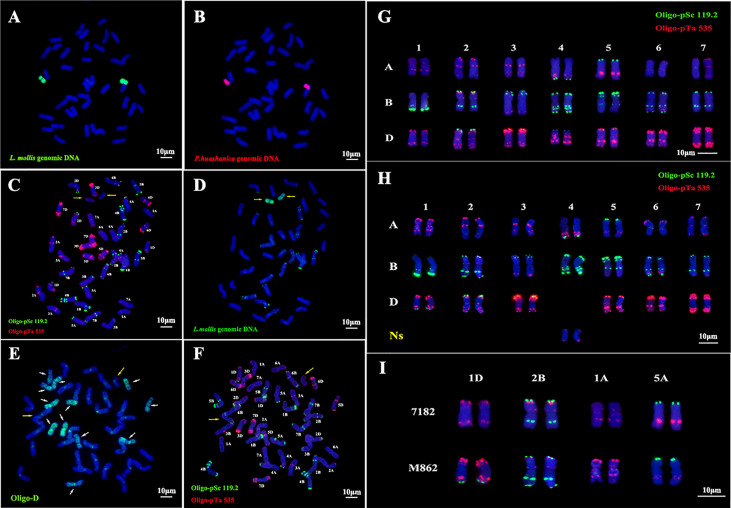
*In situ* hybridization identification of wheat-*Leymus mollis* substitution line M862. **(A)** GISH analysis of M862 using *L. mollis* genomic DNA as the probe (green). **(B)** Sequential GISH analysis of M862 using *P. hushanica* genomic DNA as the probe (red). **(C)** mc-FISH analysis of M862 using probes Oligo-pSc119.2 (green) and Oligo-pTa535 (red). **(D)** GISH analysis of M862 using *L. mollis* genomic DNA as the probe (green). **(E)** FISH analysis of M862 using Oligo-D probe (green), the white arrow referred 12 D chromosomes. **(F)** Sequential FISH analysis of M862 using probes Oligo-pSc119.2 (green) and Oligo-pTa535 (red). **(G)** FISH karyotype of parent 7182 using probes Oligo-pSc119.2 (green) and Oligo-pTa535 (red). **(H)** FISH karyotype of M862 using probes Oligo-pSc119.2 (green) and Oligo-pTa535 (red). **(I)** FISH signal comparison between CS, 7182, and M862 on chromosomes 1D, 2B and 6B. The alien chromosomes are indicated by yellow arrows. Chromosomes were counterstained with DAPI (blue). Scale bar = 10 μm.

GISH was employed to analyze the source of alien chromosomes for M862 at mitotic metaphase by labeled genomic DNA of *L. mollis* and *P. huashanica*. It was observed that two chromosomes had the green signal as evidence for *L. mollis* (**Figure 2A**). On the same slide, the two *L. mollis* chromosomes were labeled by the red signal of *P. huashanica* (**Figure 2B**). The remaining 40 chromosomes labeled with a blue signal were derived from wheat. These results indicated that the two alien chromosomes were derived from *L. mollis* Ns genome.

Sequential FISH-GISH results showed that the 4D chromosomes were absent and two chromosomes of *L. mollis* had weak red signal labeled with Oligo-pTa535, while there were no hybridization signals on alien chromosomes when labeled with Oligo-pSc119.2 (**Figures 2C, D**). 12 D-genome chromosomes were labeled with the green signal in M862 using Oligo-D (**Figure 2E**). ND-FISH analysis further indicated that two D chromosomes were missed (**Figure 2F**). Therefore, two 4D chromosomes were replaced by two Ns chromosomes of *L. mollis* in M862.

Oligo-pSc119.2 (green) and Oligo-pTa535 (red) were used to construct the FISH karyotype of 7182 (**Figure 2G**) and M862 (**Figure 2H**). The chromosome structural variation of M862 was analyzed based on the 7182 FISH karyotype (**Figures 2H, I** **)**. It was found that the end of 1DL and 2BL chromosomes were labeled with the green signal in M862, whereas the 2BS chromosome of both 7182 and M862 was labeled with the red signal. In addition, Oligo-pTa535 probe signal intensity differences could be seen in 1AS (much stronger) and 5AL (much weaker) in M862. These results suggest that the alien substitution of wheat 4D chromosomes may be accompanied by chromosome structures mutations in chromosomes 1A, 1D, 2B and 5A.

### 3.3 Analysis of foreign chromosome homoeologous group in M862

**Figure 3:**
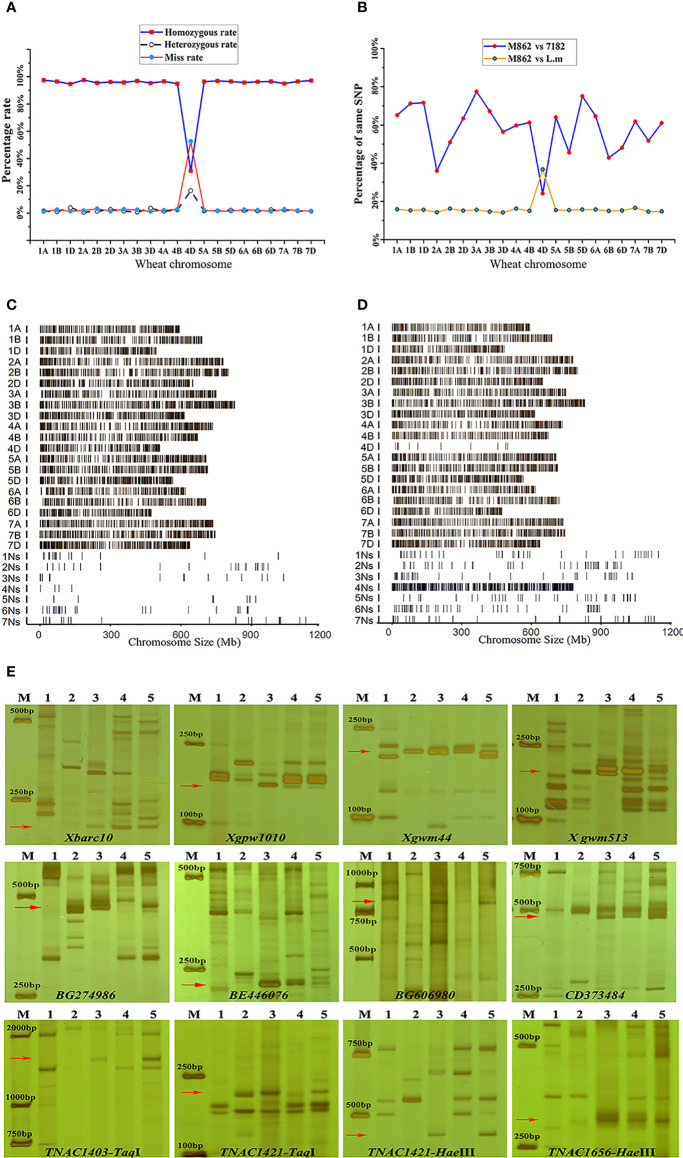
Molecular marker analysis of wheat-*Leymus mollis* substitution line M862. **(A, B)** Wheat 55K array analysis. **(A)** Obvious heterozygous and deletions on the 4D chromosome. **(B)** Obvious crossing point in terms of the position of the 4D chromosome. **(C, D)** Wheat-*P. huashanica* liquid array (GenoBaits^®^WheatplusPh) analysis. **(C)** The genomic sequence of the wheat parent line 7182 is the main distribution on chromosomes 1A-7D. **(D)** The sequences of M862 are rare distribution in 4D, but intensively distributed in 4Ns. **(E)** SSR, EST and PLUG markers analysis. M: D2000, 1: Wheat parent line 7182; 2. P*. huashanica*; 3: *L. mollis*; 4: Wheat-*L. mollis* partial amphidiploid line M47; 5: M862. The x-axis refers to the position of markers on wheat in **(A)** and **(B)**. The red arrow represents the specific band of the 4Ns chromosome of *L. mollis* or *P. huashanica*.

The chromosome composition of M862 was assessed by wheat 55K SNP array. A total of 51256, 51531 and 30076 SNP loci were detected in M862, 7182 and *L. mollis*, respectively (**Supplementary Table 2**). The miss rate (52.5%), homozygous rate (31.1%) and heterozygous rate (16.4%) of SNP in 4D chromosome were substantially obviously higher than those in other chromosomes (**Figure 3A**). Correspondingly, the maximum percentage of SNP loci shared between M862 and *L. mollis* was on 4D chromosome (36.8%), which also displayed the minimum percentage of SNP loci shared between M862 and 7182 (24.1%) (**Figure 3B**).

GenoBaits^®^WheatplusPh, a liquid array contained 90000 capture probes derived from both wheat (10000 capture probes) and *P. huashanica* (80000 capture probes), displayed in a way that could be understood more intuitively. For 7182, the detecting signal was evenly distributed along 21 wheat chromosomes and showed low signal intensity along seven *P. huashanica* chromosomes (1Ns-7Ns) (**Figure 3C**). In contrast, detecting signals in M862 occurred quite rarely on wheat 4D chromosome, but were significantly enriched on the *P. huashanica* 4Ns chromosome (**Figure 3D**).

A total of 169 molecular markers in the fourth homoeologous group were used to verify the accuracy of chip results. We observed that 36 (21.30%) markers amplified the same special bands in M862 and *L. mollis*, including 15 (12.30%) SSR makers, 10 (29.41%) EST-STS makers and 11 (84.62%) PLUG makers. Among them, 14 (38.89%, 14/36) amplified the same bands among M862, *L. mollis* and *P. huashanica* (**Figure 3E**; **Supplementary Table 3**). The remaining 22 markers displayed different amplification products between *L. mollis* and *P. huashanica*, indicating that there were some differences between the Ns chromosomes of *L. mollis* and *P. huashanica*. Overall, the molecular marker and high-throughput assay further confirmed that M862 was a wheat-*L. mollis* 4Ns(4D) alien disomic substitution line.

### 3.4 Evaluation of disease resistance and agronomy traits for M862

**Figure 4:**
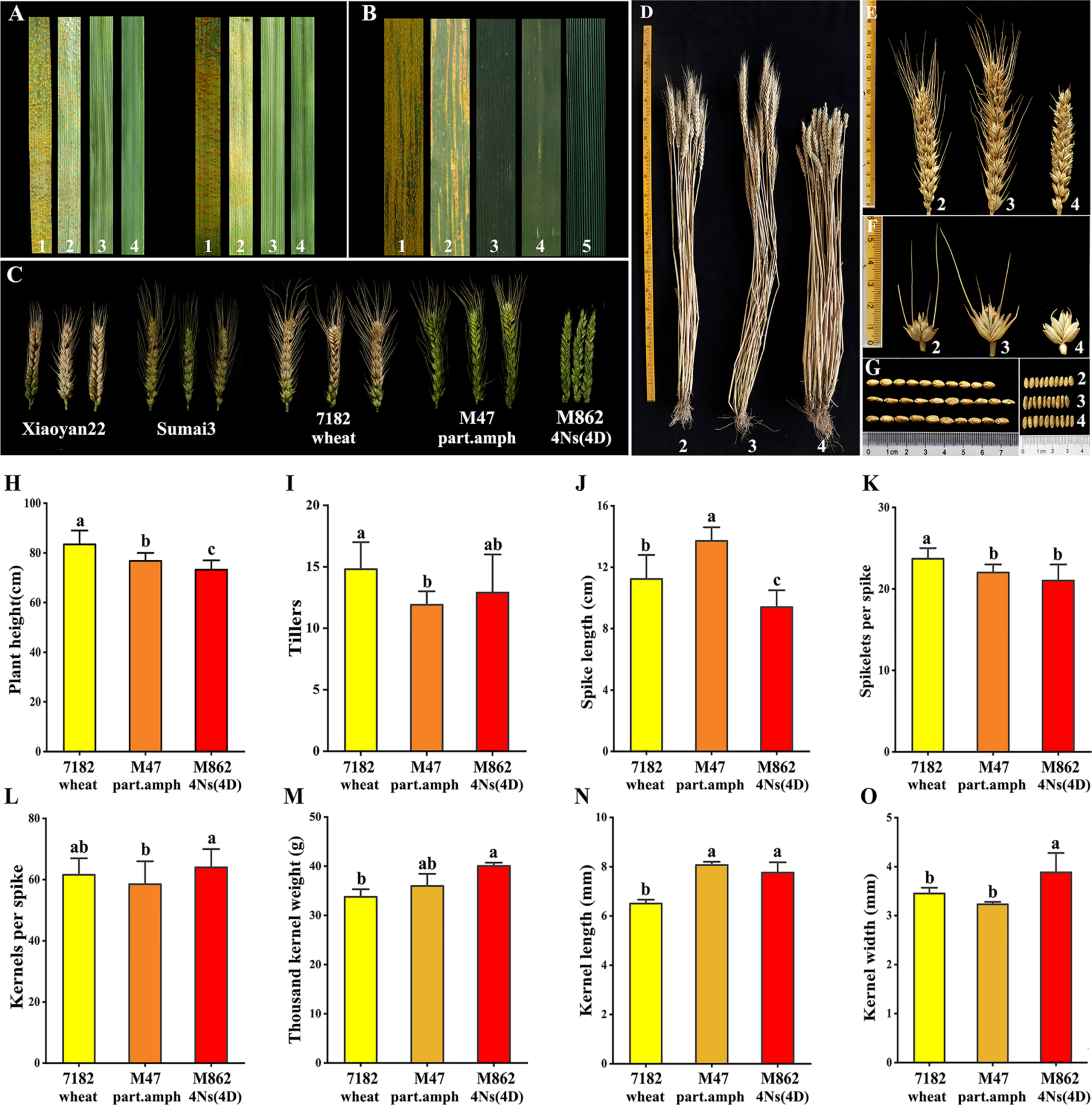
Evaluation of disease resistance and agronomic traits. **(A, B)** Identification of Stripe rust resistance of M862 and its parents. **(A)** Seedling stage reactions to *Pst* race CYR32 (left) and CYR34 (right). **(B)** Adult stage reactions with mixture *Pst* race CYR32 and CYR33. **(C)** FHB reactions to F.g PH1 of M862 and its parents. **(D-O)** Analysis of agronomic traits of M862 and its parents (*p* < 0.05). **(D)** Adult plant. **(E)** Spikes. **(F)** Spikelets. **(G)** 10-seeds length and width. 1: HXH; 2: 7182 wheat; 3: M47 part. amp; 4: M862 4Ns(4D); 5: *L. mollis*. **(H)** Plant height. **(I)** Tillers. **(J)** Spike length. **(K)** Spikelets per spike. **(L)** Kernels per spike. **(M)** Thousand kernel weight. **(N)** Kernel length. **(O)** Kernel width.

M862 and its parents were inoculated with CYR32 and CYR34 *Pst* races to determine stripe rust resistance at seedling stage. Half a month after inoculation, the susceptible control HXH had become fully diseased. Three successive investigations of disease resistance showed that 7182 was susceptible to CYR32 and CYR34. Nevertheless, the immunity type of M862 to CYR32 and CYR34 was corresponding to 0 and 2, indicating immunity and middle resistance, respectively. Correspondingly, its parents (M47) displayed high-level resistance to CYR32 and immunity to CYR34 (**Figure 4A**; **Supplementary Table 4**).

At the adult stage, the mixed *Pst* races CYR32, CYR33 and CYR34 were used to assess M862 and its parents over two growing seasons (2021, 2022). The control HXH was highly susceptible to the mixed *Pst* races CYR32 and CYR33 (**Figure 4B**; **Supplementary Table 4**), displaying a large number of visible uredia and a lack of visible cell death. That of 7182 was moderate sensitivity. However, *L. mollis* and its derived materials (M47 and M862) showed high resistance, with very few or no uredia and prominent cell death. The immunity types of M47 and M862 were 0 and 1, respectively. These results demonstrated that M862 carried new stripe rust resistance genes from *L. mollis* and was effective at the seedling and the adult stages, indicating 4Ns chromosome of *L. mollis* may carry a broad spectrum of stripe rust resistance genes.

After two consecutive years (2021 and 2022) of FHB susceptibility identification, the results of all materials were relatively consistent. The control XY22 and SM3 were susceptible and resistant to FHB, respectively. The infected spikelet rate (ISR = 3.45%, 10.50%) and severity level (SL =1, 1.4) of M862 were significantly lower than the parent 7182, but it was similar to the parent M47 (**Figure 4C**; **Supplementary Table 5**). The results showed that M862 inherited resistance gene(s) of FHB from M47, and finally traced back to the resistance source of *L. mollis*.

The agronomic traits were evaluated for M862 and its parents 7182 and M47 (**Figures 4D–O**). For M862, the plant height and spike length were significantly shorter than its parents, whereas the tillers and number of kernels per spike were similar to its parents. M862 had more 1000-kernel weight and wider kernel width than its parents. The kernel length of M862 was similar to M47 but longer than 7182. M862 had ultrashort awn, which was different from M47 and 7182. In conclusion, M862 is a potential material for increasing yield, because of its excellent properties including increased 1000-grain weight and kernel width, and keeping other traits constant.

### 3.5 Impact of Lm#4Ns on wheat gene expression

**Figure 5:**
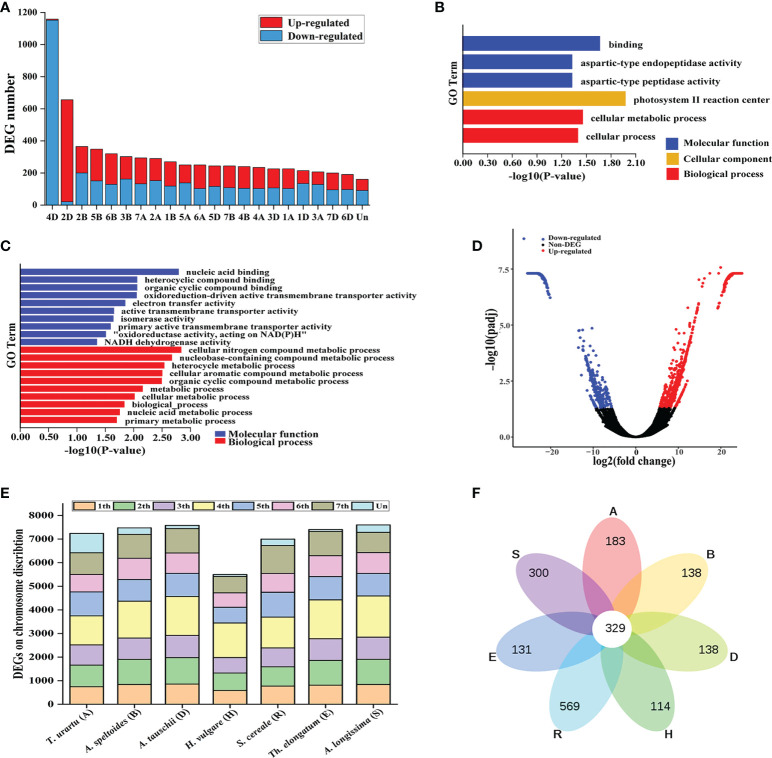
Characterization of Lm#4Ns candidate genes using transcriptome sequencing. **(A)** The chromosome distribution of DEGs between M862 and 7182. **(B)** Enrichment of up-regulated genes using Gene Ontology (GO). **(C)** The top 10 enriched GO terms for down-regulated genes. **(D)** Volcano plot indicating candidate genes for Lm#4Ns. **(E)** The distribution of Lm#4Ns candidate genes among each homologous chromosome group. **(F)** Venn-diagram of Lm#4Ns candidate genes have orthologs located on chromosomes of homoeologous group 4 among related species. *T. urartu* (A), *A speltoides* (B), *A tauschii* (D), *H vulgare* (H), *S. cereale* (R), *Th. elongatum* (E) and (*A*) *longissima* (S) genomes.

To investigate the impact of the Lm#4Ns on gene expression, we compared wheat gene expression profiles between leaf (3-leaf seedling stage) of 7182 and M862 using transcriptome sequencing. After removing low-quality reads, a total of 1.94 × 10^8^ paired reads were obtained, with an average of 10.19 G clean bases for each sample (**Supplementary Table 6**). On average, approximately 89.6% of reads were aligned to the Chinese Spring genome (IWGSC-RefSeq-v1.1), supporting the expression evidence for 37493 high-confidence wheat genes (FPKM ≥ 1 in at least one sample). Expression analysis revealed a total number of 6894 significant differentially expressed genes (DEGs) (adjusted *p*≤ 0.05; Log_2_|fold change| ≥ 1) (**Supplementary Table 7**). Among them, 3644 genes (52.86%) were down-regulated in M862, of which 1152 genes (31.61%) were distributed on chromosome 4D (**Figure 5A**; **Supplementary Table 8**). Compared to 7182, the substitution of 4D chromosome by Lm#4Ns in M862 would up-regulated the expression of 3250 genes, which was more highly distributed on chromosome 2D (635 up-regulated genes, 19.54%).

Gene ontology (GO) analysis showed that the 1531 up-regulated genes were enriched in molecular function mainly related to aspartic-type endopeptidase activity, and cation transmembrane transporter activity (**Figure 5B**; **Supplementary Table 9**). Meanwhile, the 3363 down-regulated genes were significantly enriched in nucleic acid binding, heterocyclic compound binding, organic cyclic compound binding, transition metal ion binding, cellular nitrogen compound biosynthetic process, and biosynthetic process (**Figure 5C**, **Supplementary Table 10**). Overall, these results indicated that the substitution of 4D chromosome by Lm#4Ns had a certain effect on wheat gene expression and was involved in a subset of biological processes.

### 3.6 Development and evaluation of Lm#4Ns chromosomes specific molecular markers

To create a reference transcriptome for Lm#4Ns, the unmapped reads from all six samples were then assembled using Trinity software (v2.8.4). We obtained a total of 208,181 trinity unigenes, with an average length of 817 bp (**Supplementary Table 6**). A total of 12,895 differentially expressed unigenes were found between M862 and 7182, including 7249 up-regulated genes and 5647 down-regulated genes (**Figure 5D**; **Supplementary Table 11**). Among them, 4117 unigenes (31.9%) could be annotated with the Swiss-Prot database (**Supplementary Table 12**). To investigate the homology between Lm#4Ns and wheat related species, all the 12,895 unigenes were searched against the reference genome sequence of *T. urartu* (A), *A. speltoides* (B), *A. tauschii* (D), *H. vulgare* (H), *S. cereale* (R), *Th. elongatum* (E) and A. *longissima* (S) using local BLAST. The distribution of mapping numbers ranged from 5948 to 7600 for all the comparisons (**Figure 5E**; **Supplementary Table 13**). Approximately 26.7% (3,443/12,895) of these unigenes had the highest identity to the ones on the fourth chromosome group in at least one related species (**Figure 5F**; **Supplementary Table 13**). A. *longissima* (S) (13.53%) had the highest number of hits, followed by E (12.77%), D (12.77%), B (12.12%), H (11.37%), R (10.14%) and A (9.55%).

**Figure 6:**
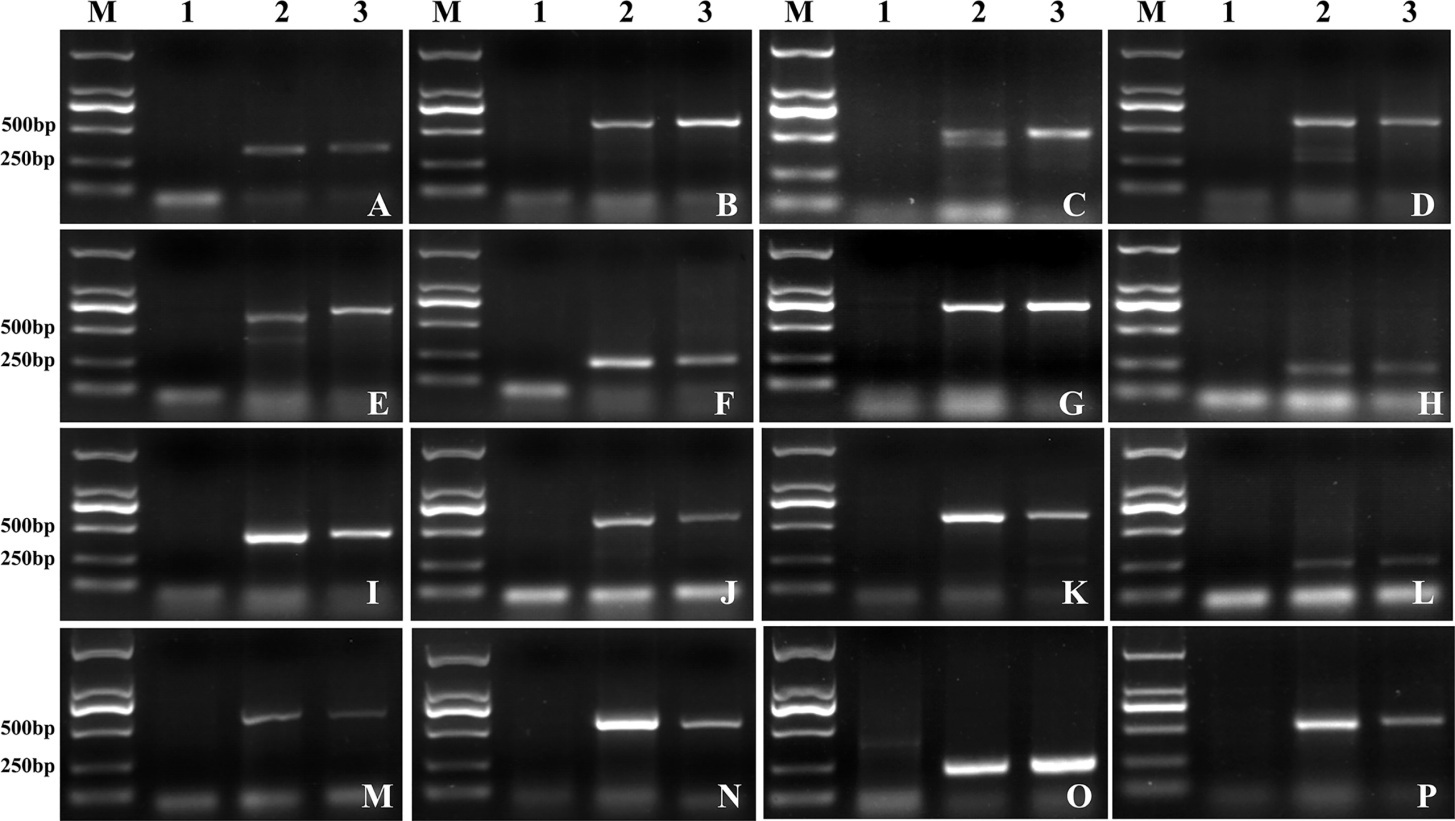
Candidate special primers for chromosomes of Lm#4Ns were designed by RNA-seq DEGs. **(A)** *Lm4-2*. **(B)** *Lm4-3*. **(C)** *Lm4-4*. **(D)** *Lm4-5*. **(E)** *Lm4-6*. **(F)** *Lm4-7*. **(G)** *Lm4-10*. **(H)** *Lm4-15*. **(I)** *Lm4-17*. **(J)** *Lm4-22*. **(K)** *Lm4-26*. **(L)** *Lm4-29*. **(M)** *Lm4-36*. **(N)** *Lm4-37*.**(O)** *Lm4-42*. **(P)** *Lm4-43*. M: D2000, 1: 7182, 2: *L. mollis*, 3: M862.

Additionally, a total of 458 Lm#4Ns unigenes were selected following the criteria as described in the methods, which were used to develop potential suitable markers. 449 Lm#4Ns candidate markers were designed, with a successful primer designing potential of 98.03% (**Supplementary Table 14**). The validation of newly developed Lm#4Ns markers was performed using 50 randomly selected markers. Finally, sixteen (32%) markers had similar amplification products in both M862 and *L. mollis*, but were absent in 7182 (**Figure 6**; **Supplementary Table 15**). These markers were considered as candidate-specific markers for tracing Lm#4Ns chromosomes in wheat background.

**Figure 7:**
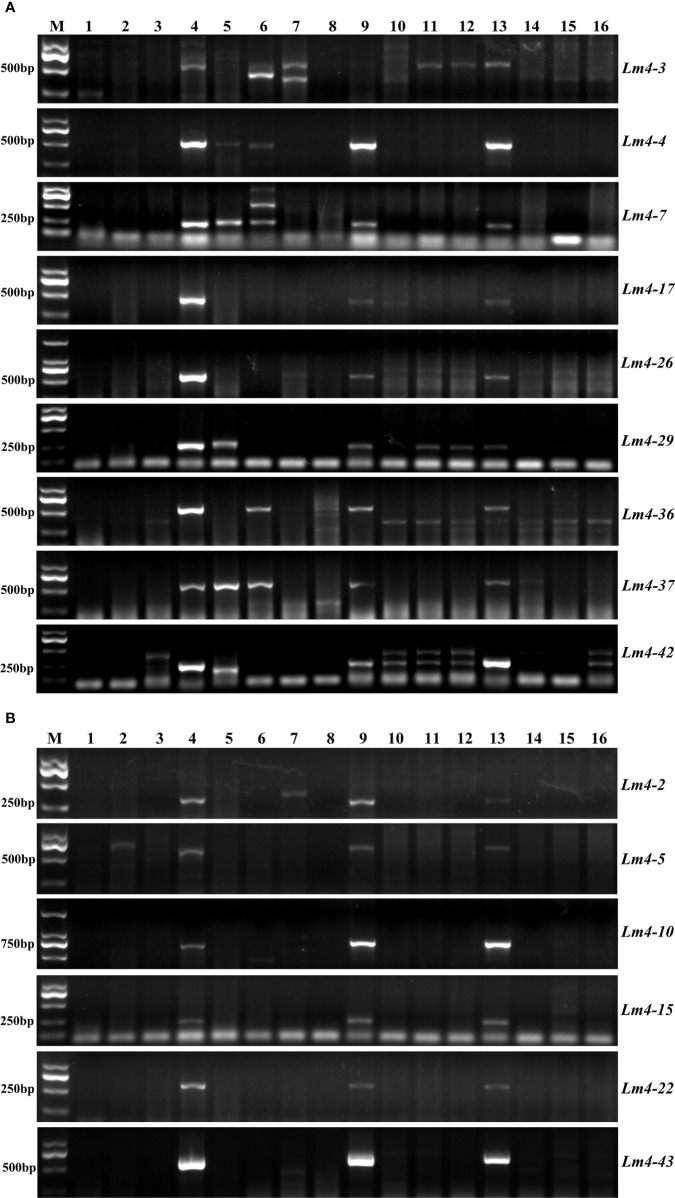
The amplification of Lm#4Ns candidate specific primers in wheat, wheat-related species, and different chromosomes of *L. mollis*. **(A)** The target bands were amplified on different species or on other chromosomes of *L. mollis.* **(B)** The target bands were amplified only on the Lm#4Ns. M: D2000; 1: CS; 2: AK58; 3: 7182; 4: *L. mollis*; 5: *P. huashanica*; 6: *Th. ponticum*; 7: *S. cereale*; 8: *H vulgare*; 9: M47; 10: Lm#1Ns(1D); 11: Lm#2Ns; 12: Lm#3Ns; 13: Lm#4Ns(4D); 14: Lm#5Ns; 15: Lm#6Ns; 16: Lm#7Ns.

To evaluate the specificity of obtained markers for Lm#4Ns chromosomes, these 16 markers were further amplified in common wheat (CS and AK58), wheat related species (*P. huashanica*, *Th. ponticum*, *S. cereale*, *H. vulgare*) and wheat-*L. mollis* derived lines (Lm#1Ns(1D), Lm#2Ns, Lm#3Ns, Lm#5Ns, Lm#6Ns and Lm#7Ns). We observed that seven markers (*Lm4-2*, *Lm4-5*, *Lm4-6*, *Lm4-10*, *Lm4-15*, *Lm4-22*, *Lm4-43*) were only amplified in *L. mollis* and its derivative lines (M862 and M47) (**Figure 7A**; **Supplementary Table 15**), which could be used to trace Lm#4Ns chromosome. The remaining 9 markers showed polymorphism between Lm#4Ns and other materials (**Figure 7B**; **Supplementary Table 15**). Among them, five markers displayed transferable to *P. huashanica* and *Th. ponticum*, of which 3 markers (*Lm4-4*, *Lm4-7*, *Lm4-37*) showed similar product size. The markers *Lm4-29* and *Lm4-42* amplified specific bands in *P. huashanica*. The other two markers (*Lm4-3*, *Lm4-36*) amplified the same bands among *Th. ponticum*, *S. cereale* and *H. vulgare*. In addition, two markers (*Lm4-3* and *Lm4-26*) and marker *Lm4-36* amplified specific bands for *S. cereale* and *H. vulgare*, respectively. Overall, these 16 newly developed markers were useful resources for tracing Lm#4Ns chromosomes and other Triticeae species during molecular breeding.

## 4 Discussion

*In situ* hybridization is a visual and accurate technique that is widely used in wheat distant hybridization (Metzlaff et al., 1986; Rayburn and Gill, 1987; Yang et al., 2015; Zhang et al., 2017a; Li et al., 2021). Genomic DNA of *L. mollis* and *P. huashanica* were usually used as GISH probe to identify the introduced *L. mollis* chromosome in wheat background (Yang et al., 2014a; Zhang et al., 2017a; Feng et al., 2022). In this study, the combination of GISH and FISH analysis demonstrated that the alien chromosomes in M862 were obtained from the *L. mollis* Ns genome, supporting that M47 parental amphidiploid line contains chromosomes from only Ns genome (Yang et al., 2014a). Since no FISH karyotype has been published in *L. mollis*, we performed wheat 55K SNP arrays to further confirm the composition of M862 chromosomes. Meanwhile, it was the first time that the wheat-*P. huashanica* liquid array (GenoBaits^®^WheatplusPh) was used to trace the foreign chromosome of wheat-*L. mollis* derivates. GenoBaits^®^WheatplusPh, a genotyping by target sequencing (GBTS) platform through in-solution capture, is constituted by 90000 capture probes derived from both wheat (10000 capture probes) and *P. huashanica* (80000 capture probes). Finally, the accuracy of the liquid array was further verified by combining the molecular markers of the fourth homoeologous group of wheat. Results showed that the GenoBaits^®^WheatplusPh displayed in a way that could be understood more intuitively to characterize the chromosome composition of wheat-*L. mollis* derivates. By taking advantage of these high-throughput platforms, their utilization will make a great contribution to chromosome engineering breeding.

The introduction of alien chromosomes into wheat by distant hybridization has occurred homoeologous chromosomal recombination between the wheat chromosomes and those of alien chromosomes (Han et al., 2004; Wang et al., 2020c). For example, Zhong 1 had a chromosome number of 52 that included 10 alien chromosomes and 42 wheat chromosomes, of which one pair of its A subgenome chromosomes contained a terminal *Th. intermedium* segment on the short arm (Han et al., 2004). Our earlier study showed that the substitution of chromosome 1D by *Th. ponticum* 1J^s^ in common wheat 7182 was accompanied by chromosome structure mutations in wheat chromosomes 5A and 6B (Wang et al., 2020c). In our study, a comparison of FISH karyotype between M862 and its wheat parent line 7182 indicates that chromosome structure mutations may have occurred in chromosomes 1A, 1D, 2B, and 5A. These observations suggest that the presence of *L. mollis* 4Ns chromosome may suppress the wheat homoeologous pairing control genes, and hence enhance limited homoeologous chromosomal recombination between wheat chromosomes and those of *L. mollis*. Further transcriptome analysis was performed to assess the effects of chromosomal recombination on gene expression. Ours results revealed that the expression of 18.39% wheat expressed genes were significantly altered in M862. As expected, wheat chromosome 4D held the highest number of DEGs and majority of them were down-regulated. However, we did not observe obvious DEGs distributed on 4A and 4B chromosomes, suggesting that Lm#4Ns may achieve the best compensatory effect for the absence 4D chromosome after multiple generations of continuous self-crossing. Additionally, the number of DEGs on chromosome 2B ranks the third, but few DEGs are distributed on chromosome 1A, 1D and 5A. By combining the altered karyotypes of 1A, 1D, 2B and 5A in M862, we infer that the structural variation of 1D, 1A and 5A may have smaller impact on gene expression than that for 2B.

Stripe rust and FHB are two major diseases threatening wheat production worldwide. The limited availability of resistance genes in cultivated wheat and the continuous evolution of new pathogen races make it necessary to constantly search for new resistance sources (Li et al., 2020). To date, a series of germplasms with resistance to stripe rust at the adult stage have been developed from the distant hybridization between wheat and *L. mollis*, including two partial amphidiploids (M47 and M42) (Yang et al., 2014a), two translocation lines (shannong0096 and WL24-4) (Bao et al., 2012; Li et al., 2015), four disomic addition liens (2Ns, 3Ns, 6Ns and 7Ns) (Li et al., 2016; Yang et al., 2017; Du et al., 2020; Yang et al., 2020), and a 7Ns(7D) alien disomic substitution line (Yang et al., 2015). For FHB, a few wheat-*Leymus* derivatives have been identified as resistance resource (Chen et al., 2005). For example, three alien introgressions sharing a common distal segment of 7Lr#1S designated a novel FHB resistance gene *Fhb3* (Qi et al., 2008), a translocation line T3AS-Lr7S (Wang et al., 2009) and a double substitution line DM96 (Lm#2Ns+3Ns) (Zhao et al., 2019) were highly resistant to FHB. According to previous studies, 2Ns, 3Ns, 6Ns and 7Ns chromosomes of *L. mollis* have stripe rust resistance, while the terminal of 7Lr of *Leymus* with *Fhb3* have FHB resistance. In this study, M862 showed high resistance to stripe rust at the seedling and adult stages, as well as high resistance to FHB. Further analysis demonstrated that the resistance genes originated from Lm#4Ns, indicating that M862 is a novel resistance source of stripe rust and FHB. Particularly, M862 displayed lower plant height, enhanced 1000-grain weight, wider grain width, and keeping other traits constant. These results suggest that M862 could be exploited as a useful intermediate material in wheat improvement and breeding.

It remains challenging to *de novo* assemble the transcriptome of most Triticeae species and further utilization of transcriptome sequencing in developing species-specific markers in wheat background (Li et al., 2019b). For example, 33 EST markers on the 6P chromosome of *Agropyron cristatum* (Dai et al., 2012), 75 EST-SSR genome-specific molecular markers of *Thinopyrum intermedium* (Cui et al., 2016), 25 *Dasypyrum villosum* 6V#4S−specific markers (Li et al., 2017), 134 *Aegilops longissima* specific markers (Wang et al., 2018) and 76 markers specific to each chromosome arm of D. villosum#4 (Li et al., 2019b) were developed using transcriptome data. In this study, we successfully screened out 16 markers that could be used to track Lm#4Ns chromosome. Among them, seven were Lm#4Ns specific markers, and the other nine could trace the genes of *P. huashanica*, *Th. ponticum*, *S. cereale*, and *H. vulgare* to varying degrees. These markers will provide an efficient way to track the 4Ns chromosome of *L. mollis* and accelerate the process of wheat breeding.

## Data availability statement

The sequencing data from transcriptome and Wheat-*P. huashanica* Liquid Array analysis have been submitted to the National Genomics Data Center (NGDC) of the China National Center for Bioinformation (CNCB) study accession CRA007625. All data supporting the findings of this study are available within the paper and within its supplementary materials published online. The datasets and materials used during current study are also available by emailing the corresponding authors on reasonable request.

## Author contributions

The experiment was designed by WJ and PD. The experimental materials were developed by JZ, CW, CC and ZT. PD, XD and YJ analyzed the transcriptome data. PD and XD developed the molecular markers. XD and RL analyzed foreign chromosome using molecular markers. XD, RL, XF, LS evaluated materials disease resistance and agronomy traits. XD and RL validated Lm#4Ns chromosomes specific molecular marker. The manuscript was drafted by XD, WJ, PD and TL. All authors read and approved the final manuscript.

## Funding

This work was supported by the National Key Research and Development Program of China (2021YFD1200601-04) and Natural Science Basic Research Plan in Shaanxi Province of China (2022JQ-172).

## Acknowledgments

We are grateful to Prof. Xiangqi Zhang for providing materials and Prof. Gourong Wei for providing stripe rust pathogens.

## Conflict of interest

The authors declare that the research was conducted in the absence of any commercial or financial relationships that could be construed as a potential conflict of interest.

## Publisher’s note

All claims expressed in this article are solely those of the authors and do not necessarily represent those of their affiliated organizations, or those of the publisher, the editors and the reviewers. Any product that may be evaluated in this article, or claim that may be made by its manufacturer, is not guaranteed or endorsed by the publisher.
