# Supplementary figures and images for: Cytogenetic identification and molecular marker development of a novel wheat-Leymus mollis 4Ns(4D) alien disomic substitution line with resistance to stripe rust and Fusarium head blight
Source: Front Plant Sci. 2022 Nov 1;13:1012939. doi: 10.3389/fpls.2022.1012939 (PMC9667194; doi:10.3389/fpls.2022.1012939)

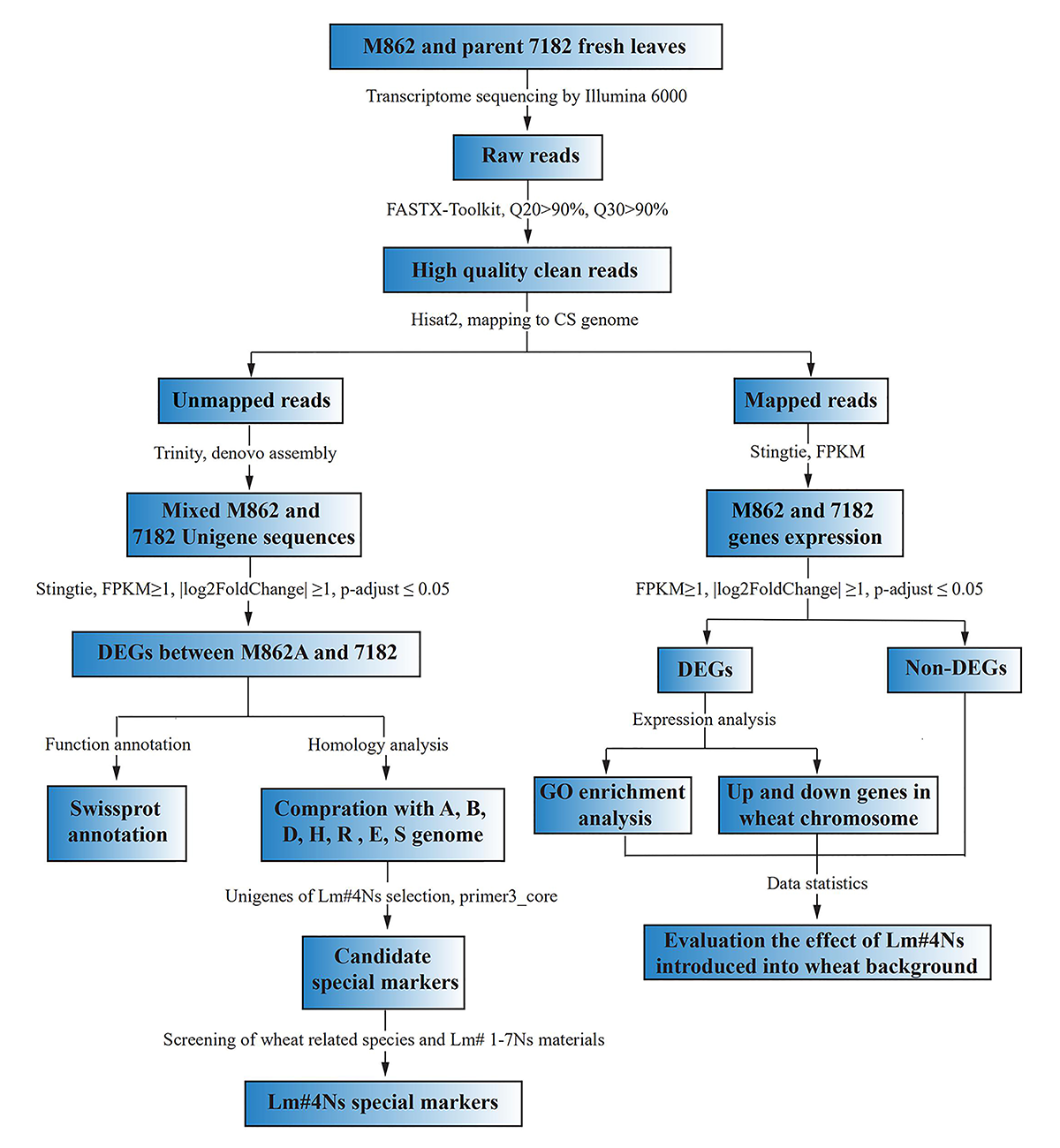

Supplement: Supplementary file 1 [file Image_1.tif]
